# Does the CCR5-Δ32 mutation explain the variable coronavirus-2019 pandemic statistics in Europe?

**DOI:** 10.3325/cmj.2020.61.525

**Published:** 2020-12

**Authors:** Nada Starčević Čizmarević, Marin Tota, Smiljana Ristić

**Affiliations:** 1 Department of Medical Biology and Genetics, Faculty of Medicine, University of Rijeka, Rijeka, Croatia *nadasc@medri.uniri.hr*; 2 Department of Medical Chemistry, Biochemistry and Clinical Chemistry, Faculty of Medicine, University of Rijeka, Rijeka, Croatia

We have read with great interest several recently published articles on the potential relevance of some genetic polymorphisms to SARS-CoV-2 infection and the severity of consequent coronavirus disease 2019 (COVID-19) (1-4). Given that the COVID-19 pandemic shows important geographic variation in its prevalence and mortality, Delanghe et al (1) hypothesized that genetic variation of the host might, at least in part, explain the outcome of SARS-CoV-2 infection. They analyzed the geographic variation of a number of immune system-related human plasma protein polymorphisms and reported that the ACE1*D allele frequency correlated negatively with COVID-19 prevalence and positively with COVID-19-associated mortality. The other investigated polymorphisms, including the F and S alleles of complement C3, the C282Y mutation in homeostatic iron regulator HFE, the Hp1 and Hp2 alleles of haptoglobin, and the DBP1 and DBP2 alleles of vitamin D binding protein, did not significantly correlate with COVID-19 prevalence or mortality (2).

Another gene that shows significant geographic variation and that regulates various aspects of the adaptive immune response is chemokine receptor 5 (CCR5). CCR5 is a receptor for proinflammatory chemokines that play key roles in host responses, especially to viruses. Furthermore, CCR5 expressed on various cell types plays a vital role in the inflammatory response by directing cells to sites of inflammation. The 32-bp deletion mutation in the CCR5 coding region (CCR5-Δ32) prevents receptor expression on the cell surface and leaves homozygous individuals without functional CCR5.

It is well known that the CCR5-Δ32 mutation prevents HIV transmission and delays the onset of AIDS. In contrast to AIDS, homozygosity for the CCR5-Δ32 allele is a strong risk factor for symptomatic West Nile virus infection and correlates with severe tick-borne encephalitis virus symptoms. It has also been shown that CCR5-Δ32 patients are at higher risk than the general population for a fatal outcome in influenza infection, and that CCR5-knockout mice have an increased mortality rate after influenza virus infection (5,6).

The CCR5-Δ32 mutation is highly prevalent in European populations, with an average frequency of 10%, and it shows a strong geographic north-to-south cline: the highest frequencies are in Nordic countries and the lowest in Southern European populations. The CCR5-Δ32 allele frequency in Croatia of 7% fits the observed European north-to-south gradient (7).

The variable prevalence of COVID-19 can be attributed to different factors (country's economic situation and levels of health services, number of diagnostics tests performed, comorbidities in a large number of fatal cases, differences in population density, demographic age distribution, environmental factors, etc), but an important factor underlying variable pandemic statistics is the influence of predisposing host genetics on susceptibility to SARS-CoV-2 infection and COVID-19.

Therefore, we compared CCR5-Δ32 mutation frequency in 39 European countries (8) with the prevalence and mortality of COVID-19, as of June 1, 2020. Data from Albania, Austria, Belarus, Belgium, Bosnia and Herzegovina, Bulgaria, Croatia, the Czech Republic, Denmark, Estonia, Finland, France, Germany, Greece, Hungary, Iceland, Italy, Latvia, Lithuania, Luxembourg, Moldova, Malta, Montenegro, the Netherlands, North Macedonia, Norway, Poland, Portugal, Romania, Russia, Serbia, Slovakia, Slovenia, Spain, Sweden, Switzerland, Turkey, the United Kingdom, and Ukraine were included in the analysis. Data on the prevalence (number of cases/10^6^ inhabitants), mortality (number of deaths/10^6^ inhabitants), number of diagnostic tests per 10^6^ people, and time elapsed since the onset of the epidemic (days since January 1, 2020) in each country were obtained from the Worldometer website (https://www.worldometers.info/coronavirus/#countries).

Multiple regression analyses revealed that the partial correlation coefficients of the CCR5-Δ32 mutation frequency showed no significant associations with the log prevalence (partial r = −0.004, *P* = 0.979) and log-mortality (partial r = −0.156, *P* = 0.355) of COVID-19, after adjusting for the log-number of diagnostic tests and log-onset of the epidemic (days) in each country as possible confounders.

Our findings did not show that the CCR5-Δ32 mutation can explain the variable prevalence and mortality of COVID-19 in European countries. Our research is limited to a well-defined area of Europe, where populations have a similar genetic background, where the frequency of CCR5-Δ32 mutation varies significantly, and where there are no major differences in other variables, such as biological, environmental, and social factors that could affect the prevalence and mortality of COVID-19, as compared with Asian and African countries.

On the other hand, very recently it has been shown that critically ill COVID-19 patients treated with a *CCR5* antagonist, leronlimab, experienced reversed hyperimmune activation and inflammation, as well as reversed immunosuppression, which thereby facilitated a more effective immune response correlated with decreases in blood SARS-CoV-2 levels (9). Accordingly, future studies of the effect of the CCR5-Δ32 mutation on the clinical course of COVID-19, as well as on the therapeutic responses of patients, are warranted.

In conclusion, this is the first report on the role of the CCR5-Δ32 mutation in the COVID-19 pandemic. The results show that the CCR5-Δ32 mutation cannot be regarded as a predictor of COVID-19 prevalence or mortality in the European population. However, there are many other confounding genetic and environmental variables affecting the COVID-19 severity and even virulence of the virus that should be taken into account in further studies.
